# A comparison of univariate and multivariate gene selection techniques for classification of cancer datasets

**DOI:** 10.1186/1471-2105-7-235

**Published:** 2006-05-02

**Authors:** Carmen Lai, Marcel JT Reinders, Laura J van't Veer, Lodewyk FA Wessels

**Affiliations:** 1 Information and Communication Theory Group, Delft University of Technology, Delft, The Netherlands; 2 The Netherland's Cancer Institute, Amsterdam, The Netherlands

## Abstract

**Background:**

Gene selection is an important step when building predictors of disease state based on gene expression data. Gene selection generally improves performance and identifies a relevant subset of genes. Many univariate and multivariate gene selection approaches have been proposed. Frequently the claim is made that genes are co-regulated (due to pathway dependencies) and that multivariate approaches are therefore per definition more desirable than univariate selection approaches. Based on the published performances of all these approaches a fair comparison of the available results can not be made. This mainly stems from two factors. First, the results are often biased, since the validation set is in one way or another involved in training the predictor, resulting in optimistically biased performance estimates. Second, the published results are often based on a small number of relatively simple datasets. Consequently no generally applicable conclusions can be drawn.

**Results:**

In this study we adopted an unbiased protocol to perform a fair comparison of frequently used multivariate and univariate gene selection techniques, in combination with a ränge of classifiers. Our conclusions are based on seven gene expression datasets, across several cancer types.

**Conclusion:**

Our experiments illustrate that, contrary to several previous studies, in five of the seven datasets univariate selection approaches yield consistently better results than multivariate approaches. The simplest multivariate selection approach, the Top Scoring method, achieves the best results on the remaining two datasets. We conclude that the correlation structures, if present, are difficult to extract due to the small number of samples, and that consequently, overly-complex gene selection algorithms that attempt to extract these structures are prone to overtraining.

## Background

Gene expression microarrays enable the measurement of the activity levels of thousands of genes on a single glass slide. The number of genes (features) is in the order of thousands while the number of arrays is usually limited to several hundreds, due to the high cost associated with the procedure and the sample availability. In classification tasks a reduction of the feature space is usually performed [1,2]. On the one hand it decreases the complexity of the classification task and thus improves the classification Performance [3-7]. This is especially true when the classifiers employed are sensitive to noise. On the other hand it identifies relevant genes that can be potential biomarkers for the problem under study, and can be used in the clinic or for further studies, e.g. as targets for new types of therapies.

A widely used search strategy employs a criterion to evaluate the informativeness of each gene individually. We refer to this approach as univariate gene selection. Several criteria have been proposed in the literature, e.g. Golub et al. [8] introduced the signal-to-noise-ratio (SNR), also employed in [9,10]. Bendor et al. [4] proposed the threshold number of misclassification (TNoM) score. Cho et al. [11] compared several criteria: Pearson and Spearman correlation, Euclidean and cosine distances, SNR, mutual information and information gain. The latter was also employed by [12]. Chow et al. [6] employed the median vote relevance (MVR), Naïve Bayes global relevance (NBGR), and the SNR, which they referred to as mean aggregate relevance (MAR). Dudoit et al. [13] employed the t-statistic and the Wilcoxon statistic. In all cases, the genes are ranked individually according to the chosen criterion, from the most to the least informative. The ranking of the genes defines the collection of gene subsets that will be evaluated to find the most informative subset. More specifically, the first set to be evaluated consists of the most informative gene, the second set to be evaluated consists of the two most informative genes and the last set consists of the complete set of genes. The set with the highest score (classification performance or multivariate criterion) is then judged to be the most informative. For a set of *p *genes, this univariate search requires the evaluation of at most *p *gene sets.

Several multivariate search strategies have been proposed in the literature, all involving combinatorial searches through the space of possible feature subsets [1,14]. In contrast to the univariate approaches, which define the search path through the space of gene sets based on the univariate evaluation of genes, multivariate approaches define the search path based on the informativeness of a *group *of genes. Due to computational limitations, relatively simple approaches, such as greedy forward search strategies are often employed [5,15]. More complex procedures such as floating searches [16] and genetic algorithms have also been applied [5,17-19]. Guyon et al. [20] employed an iterative, multivariate backward search called Recursive Feature Elimination (RFE). RFE employs a classifier (typically the Support Vector Machine (SVM)) to attach a weight to every gene in the starting set. Based on the assumption that the genes with the smallest weights are the least informative in the set, a predefined number of these genes are removed during each iteration, until no genes are left. The performance of the SVM determines the informativeness of the evaluated geneset. Bo at al. [21] introduced a multivariate search approach that performs a forward (greedy) search by adding genes judged to be informative when evaluated as a pair. Recently, Geman et al. [22,23] introduced the top-scoring pair, TSP method, which identifies a single pair of predictive genes. *Liknon *[10,24] was proposed as an algorithm that simultaneously performs relevant gene identification and classification in a multivariate fashion.

The above mentioned univariate and multivariate search techniques have been presented as successfully performing the gene selection and classification tasks. The goal of this study is to validate this claim because a fair comparison of the published results is problematic due to several limitations. The most important limitation stems from the fact that the training and validation phases are not strictly separated, causing an 'Information leak' from the training phase to the validation phase resulting in optimistically biased performances. This bias manifests itself in two forms. First, there is the most severe form identified by Ambroise et al. [25]. (See also the erratum by Guyon [26]). This bias results from determining the search path through gene subset space on the *complete *dataset (i.e. also on the validation set) and then performing a cross validation at each point on the search path to select the best subset. Although this bias is a well known phenomenon at this stage, a fairly large number of publications still carry this bias in their results [6,9-12,17,20,27,28]. The second form of bias is less severe, and was elaborately described in Wessels et al. [29]. See [4,13,21] for instances of results where this form of bias is present. Typically, the training set is employed to generate a search path consisting of candidate gene sets, while the classification performance of a classifier trained on the training set and tested on the validation set is employed to evaluate the informativeness of each gene set. The results are presented as a set of (cross)validation performances – one for every geneset. The bias stems from the fact that the validation set is employed to pick the best performing gene subset from the series of evaluated sets. Since optimization of the gene subset is part of the training process, selection of the best gene subset should also be performed on the training set only. An unbiased protocol has been recently proposed by Statnikov et al. [7] to perform model selection. Here, a nested cross-validation has been used to achieve both the optimization of the diagnostic model, such as the choice of the kernel type and the optimization parameter *c *of the SVM for example, and the performance estimate of the model. The protocol has been implemented in a System called GEMS [30]. In addition to the raised concerns, the comparison between the results in available studies is difficult since the conclusions are frequently based on a small number of datasets, often the *Colon *[31] and *Leukemia *[8] datasets. See, for example [5,12,20,21,28,32]. Sometimes even the datasets employed are judged by the authors themselves to be simple and linearly separable [10,17,18,33]. Therefore, no generally applicable conclusions can be drawn.

We perform a *fair *comparison of several frequently used search techniques, both multivariate and univariate, using an unbiased protocol described in [29]. Our conclusions are based on seven datasets, across different cancer types, platforms and diagnostic tasks. Surprisingly, the results show that the univariate selection of genes performs very well. It appears that the multivariate effects, which also influence classification performance, can not be easily detected given the limited sizes of the datasets.

## Results

The focus of our work is on gene selection techniques. We adopted several univariate and multivariate selection approaches. For each dataset, the average classification error across the folds of the 10-fold outer cross-validation and its Standard deviation are reported in Tables 1 and 2. The best result for each dataset is emphasized in bold characters. For comparison the performance of three classifiers, namely Nearest Mean Classifier (NMC), Fisher (FLD) and the Support Vector Machine (SVM), is evaluated without any gene selection being performed, i.e. when the classifiers are trained with all the genes. We judge that method *A *with mean and Standard deviation of the error rate *μ*_*A *_and *σ*_*A *_is significantly better than method *B *with mean and Standard deviation of the error rate *μ*_*B *_and *σ*_*B *_when *μ*_*B *_≥ *μ*_*A *_+ *σ*_*A*_. The stars in Tables 1 and 2 indicate results that are similar when employing this rule-of-thumb. As can be observed from Tables 1 and 2, the univariate approaches are significantly better than both the multivariate approaches and cases where no gene selection was performed in two cases: *DLBCL *and *HNSCC*. In addition, univariate approaches are the best but not significantly better for the *Breast Cancer *and *CNS *datasets, and comparable to the best approach in the remaining two cases (*Leukemia *and *Prostate*). Only for the *Colon *dataset, the univariate approaches perform significantly worse than the multivariate TSP.

Employing the t-test or SNR in the univariate approaches has no effect on the error rate when employed in combination with the NMC. However, it has a significant effect in combination with the Fisher classifier. This is mainly due to the sensitivity of the Fisher classifier when the number of training objects approaches the number of selected genes during training [34]. This stems from the fact that the size of the selected gene-sets changes considerably across the folds of the gene optimization procedure, and may lead to sub-optimal gene set optimization.

Concerning the studied multivariate techniques, the base pair (BP) and forward search (FS) approaches of Bö et al. [21] are significantly worse in the majority of the datasets, with the exception of the base pair approach in the case of the *Colon *dataset. The *Liknon *classifier reaches error rates comparable to univariate results on the *CNS *and *Colon *datasets. The Recursive Feature Elimination [20] performs slightly better than the other multivariate approaches achieving performances that are not significantly worse than the best approach on four datasets. However, in three of these cases, the performance is similar to the results achieved without any gene selection. As was observed by [20], our results also indicate that there is no significant difference between RFE employing the Fisher or SVM classifiers. Although the TSP method is the best performing approach for the *Colon *and *Prostate *datasets, its performance is not stable across the remaining datasets, in fact, it is worse than the best performing method in all the remaining datasets. Summarizing, in six of the seven adopted datasets there is no detectable improvement when employing multivariate approaches, since better or comparable performances are obtained with univariate methods or without any gene selection. The classification performance alone cannot be regarded as an indication of biological relevance, since a good classification could be reached with different gene sets, and gene-set sizes, depending on the methodology employed. This is in agreement with the studies of Eindor at al. [3] and Michiels et al. [35]. These studies pointed out that the selected gene sets are highly variable depending on the sampling of the dataset employed during training. However, different gene-sets perform equally well [3,6,8,10], indicating that there is, in fact, a large collection of genes that report the same underlying biological processes, and that *the unique gene set *does not exist. The lack of performance improvement when applying multivariate gene selection techniques could also be caused by the small sample size problem. This implies that there are too few samples to detect the complex, multivariate gene correlations, if these were actually present. Only one multivariate approach, namely the TSP method, was able to extract a pair of genes that significantly improved the classification performance.

## Conclusion

In gene expression analysis gene selection is imdertaken in order to achieve a good classification Performance and to identify a relevant group of genes t hat can be further studied in the quest for biological understanding of the cancer mechanisms. In the literature it is claimed that both multivariate and univariate approaches successfiilly achieve both purposes. However, these results are often biased since the training and validation phases of the classifiers are not strictly separated. Moreover, the results are often based on few and relatively simple datasets. Therefore no clear conclusions can be drawn. Therefore, we have performed a comparison of frequently used multivariate and univariate gene selection algorithms across a wide ränge of cancer gene expression datasets within a framework which minimizes the Performance biases mentioned above.

We have found that univariate gene selection leads to good and stable performances across many cancer types. Most multivariate selection approaches do not result in a performance improvement over univariate gene selection techniques. The only exception was a significant performance improvement on the *Colon *dataset employing the TSP classifier, the simplest of the investigated algorithms employing multivariate gene selection. However, the performances of the TSP method are not stable across different datasets. Therefore, we conclude that correlation structures, if present in the data, cannot be detected reliably due to sample size limitations. Further research and larger datasets are necessary in order to validate informative gene interactions.

## Methods

### Gene selection techniques

In this section we elaborate on the different univariate and multivariate selection strategies employed in this study. The approaches are cast in a general framework which highlights the choices made by the user, and facilitates direct qualitative comparison of these approaches.

Gene selection approaches are, in fact, optimization strategies, which input

1. *D*, a dataset consisting of *n *object-label pairs,

2. *θ*_Ω_, a set of user-defined parameters which specify which type of classifier to use, and possible algorithm dependent choices such as the ranking criterion and

3. *θ*_Φ_, another user-defined parameter defining the evaluation procedure (if cross-validation is employed, would specify the number of folds) and which return the optimal value of a tunable parameter, *ϕ*, such that the gene set associated with ϕ* (the optimal value of the tunable parameter) corresponds to the most informative gene set. During this optimization process, each gene selection approach is characterized by its own unique way to traverse and evaluate various gene sets. If we denote the mapping associated with selection approach *A *by Φ_*A*_, this can be formally expressed in the following way:

*ϕ*_*A *_= Φ_*A*_(*D*, *θ*_Ω_, *θ*_Φ_).     (1)

For all the gene selection techniques described in this paper, the gene selection technique employs a classifier to evaluate the informativeness of the gene set associated with a given setting of *ϕ *. Given a dataset, *D*, and a setting of *ϕ*, the process which results in this classifier involves both a gene selection and classifier training step which could be separate or integrated. (This will be elaborated upon in the detailed descriptions of each technique). Formally, this process can be described as follows:

*ω*_*A *_= Ω_*A*_(*D*, *θ*_Ω_, *ϕ*_*A*_),     (2)

where *ω*_*A *_is the classifier trained on the geneset resulting from *ϕ*_*A*_, *θ*_Ω _represents the previously define Parameters, and Ω_*A*_(.) is a mapping representing the training and selection process. During the optimization process, Φ_*A*_(.) repeatedly calls Ω_*A*_(.) with different settings for *ϕ *and employs the Performance of *ω*_*A *_as quality measure to guide the process. Upon completion of the optimization, the optimal classifier associated with the optimal gene set is given by:

ωA*=ΩA(D,θΩ,φA*).     (3)
 MathType@MTEF@5@5@+=feaafiart1ev1aaatCvAUfKttLearuWrP9MDH5MBPbIqV92AaeXatLxBI9gBaebbnrfifHhDYfgasaacH8akY=wiFfYdH8Gipec8Eeeu0xXdbba9frFj0=OqFfea0dXdd9vqai=hGuQ8kuc9pgc9s8qqaq=dirpe0xb9q8qiLsFr0=vr0=vr0dc8meaabaqaciaacaGaaeqabaqabeGadaaakeaaiiGacqWFjpWDdaqhaaWcbaGaemyqaeeabaGaeiOkaOcaaOGaeyypa0JaeuyQdC1aaSbaaSqaaiabdgeabbqabaGcdaqadaqaaiabdseaejabcYcaSiab=H7aXnaaBaaaleaacqqHPoWvaeqaaOGaeiilaWIae8NXdy2aa0baaSqaaiabdgeabbqaaiabcQcaQaaaaOGaayjkaiaawMcaaiabc6caUiaaxMaacaWLjaWaaeWaaeaacqaIZaWmaiaawIcacaGLPaaaaaa@44B9@

### Univariate gene selection

In the univariate approach (U) the informativeness of each gene is evaluated individually, according to a criterion, such as the Pearson correlation, t-statistic or signal-to-noise ratio (SNR) [4,6,11,13]. The genes are ranked accordingly, i.e. from the most to the least informative. This ranking defines a series of gene sets as well as the order in which they are subsequently evaluated. The first gene set is the best ranked gene, the second gene set the best two ranked genes, etc. The informativeness of each gene set is evaluated by estimating its cross-validation performance in combination with a particular classifier. As ranking criterion we adopt the SNR and the t-statistic. The former, due to its simplicity and popularity [6,8,20,27,36], and the latter in order to enable a better comparison with [21]. For the evaluation of every gene set, we employ the Nearest Mean Classifier (NMC) with cosine correlation as distance measure and the Fisher classifier (FLD). The Fisher classifier [14,37] is a linear discriminant, it projects the data in a low dimensional space chosen by maximizing the ratio of the between-class and within-class scatter matrices of the dataset, and in this space classifies the samples. The within-class matrix is proportional to the pooled sample covariance matrix. In case of singularity of the matrix, which arises if the number of samples is smaller than the number of dimensions, the pseudo-inverse is used. In terms of the formal framework, *θ*_Ω _represents the choice of univariate criterion (SNR or t-statistic) and classifier, while *ϕ *represents the desired number of genes selected. For *ϕ *= *k*, this would correspond to the top *k *ranked genes. *θ*_Φ _represents the type of cross validation to employ during the training process.

### Multivariate gene selection

#### Base-pair selection (BP)

The base-pair selection algorithm was proposed for microarray datasets by Bo et al. [21]. The informativeness of genes is judged by evaluating pairs of genes. For each pair the data is first projected by the diagonal linear discriminant (DLD) onto a one-dimensional space. The t-statistic is then employed to score the informativeness of the gene pair in this space. A complete search evaluates all pairs of genes and ranks them in a list – without repetition – according to the scores. The computational complexity of this method is a serious limitation, therefore, a faster greedy search is also proposed. The genes are first ranked according to the individual t-statistic – as in univariate selection. The best gene is selected and the method searches for a gene amongst the remaining genes which, together with the individual best gene, maximizes the t-statistic in the projected space. This provides the first two genes of the ordered list. From the remaining *p – *2 genes the best individual gene is selected and matched with a gene from the remaining *p – *3 genes which maximizes the score in the projected space. This provides the second pair of genes. By iterating the process, pairs of genes are added, until all the genes have been selected. Similar to the univariate selection approach, we have now established a series of gene sets äs well as the order in which they are subsequently evaluated, once again by starting with the first pair in the ranking, and then creating new sets by expanding the previous set with the next pair of genes in the ranking. Following [21], the Fisher classifier is employed to evaluate each gene set. Formally, *θ*_Ω _represents the choice of DLD as mapping function, the t-statistic as univariate criterion in the mapped space and the choice of the Fisher classifier to evaluate the extracted gene sets. *ϕ *represents the desired number of genes to be extracted and *θ*_Φ _represents the type of cross validation to employ during gene set evaluation.

#### Forward selection (FS)

Forward gene selection Starts with the single most informative gene and iteratively adds the next most informative genes in a greedy fashion. Here, we adopt the forward search proposed by Bo et al. [21]. The best individual gene is foimd according to the t-statistic. The second gene to be added is the one that, together with the first gene, has the highest t-statistic computed in the one-dimensional DLD projected space. This set is expanded with the gene which, in combination with the first two genes, maximizes the score in the projected space – now a three-dimensional space projected to a single dimension. By iterating this process an ordered list of genes is generated, once again defining a collection of gene sets, as well as the order in which these are evaluated. Now the length of the list is limited to *n *genes. In [21] this lipper limit stems from the fact that the Fisher classifier cannot be solved (without taking additional measures) when the number of genes exceed *n*. Although elsewhere we employ the pseudo-inverse to overcome this problem associated with the Fisher classifier, we chose to maintain this lipper limit in order to remain compatible with the set-up of [21]. Moreover, it keeps the selection technique computationally feasible. The formal definition of parameters corresponds exactly to the base-pair approach, except that a greedy search strategy (instead of the approach proposed by [21]) is employed in the optimization phase.

#### Recursive Feature Elimination (RFE)

RFE is an iterative backward selection technique proposed by Guyon et al. [20]. Initially a Support Vector Machine (SVM) classifier is trained with the füll gene set. The quality of a gene is characterized by the weight that the SVM optimization assigns to that gene. A portion (a parameter determined by the user) of the genes with the smallest weights is removed at each iteration of the selection process. In order to construct a ranking of all the genes, the genes that are removed, are added at the bottom of the list, such that the gene with the smallest weight is at the bottom. By iterating the procedure this list grows from the least informative gene at the bottom, to the most informative gene at the top. Note that the genes are not evaluated individually, since their assigned weights are dependent on all the genes involved in the SVM optimization during a given iteration. As was the case in all previous approaches, a ranked gene list is produced, which defines a series of gene sets, as well as the order in which these sets should be evaluated when searching for the optimal set. In our implementation we adopt both the Fisher classifier and the SVM, with the optimization parameter set to *c *= 100 and a linear kernel. Both setups where proposed by [20]. While the Fisher classifier suffers from the dimensionality problem when *p *≈ *n *(for *p > n *regularization occurs due to the pseudo-inverse [34]), it has the advantage over the SVM that no parameters need to be optimized. Moreover, it allows for a comparison with the other studied approaches which also employ the Fisher classifier. We chose to remove one gene per iteration.

Formally, *θ*_Ω _represents the choice of SVM (or Fisher) as classifier to generate the evaluation weights for the genes, the regularization parameter of the SVM, as well as the number of genes to be removed during every iteration. *ϕ *represents the number of genes selected, while *θ*_Φ _represents the type of cross validation to employ during gene set evaluation.

#### Liknon

Bhattacharyya et al. [10,24] proposed a classifier called *Liknon *that simultaneously performs classification and relevant gene identification. Liknon is trained by optimizing a linear discriminant function with a penalty constraint via linear programming. This yields a hyper-plane that is parameterized by a limited set of genes: the genes assigned non-zero weights by Liknon. By varying the influence of the penalty one can put more emphasis on either reducing the prediction error and allowing more non-zero weights or increasing the sparsity of the hyperplane parameterization while decreasing the apparent accuracy of the classifier. The penalty term therefore directly influences the size of the selected gene set. Although [10] fixed the penalty term (*C *= 1), we chose its value in a more systematic way, via cross-validation. The penalty term was allowed to vary in the range *C *∈ [0.1,..., 100]. Formally, *θ*_Ω _is obsolete, *ϕ *represents the penalty parameter and *θ*_Φ _the choice of cross validation type.

#### Top-scoring pair

A recent classifier called *Top-scoring pair *(TSP) has been proposed by [22,23]. The TSP classifier performs a full pairwise search. Let **X **= {*X*_1_, *X*_2_,...*X*_*p*_} be the gene expression profile of a patient, with *X*_*i *_the gene expression of gene *i*. The top-scoring pair (*i, j*) is the one for which there is the highest difference in the probability of *X*_*i *_<*X*_*j *_from Class *A *to Class *B*. A new patient **X**^*d *^is classified as Class *A *if Xid<Xjd
 MathType@MTEF@5@5@+=feaafiart1ev1aaatCvAUfKttLearuWrP9MDH5MBPbIqV92AaeXatLxBI9gBaebbnrfifHhDYfgasaacH8akY=wiFfYdH8Gipec8Eeeu0xXdbba9frFj0=OqFfea0dXdd9vqai=hGuQ8kuc9pgc9s8qqaq=dirpe0xb9q8qiLsFr0=vr0=vr0dc8meaabaqaciaacaGaaeqabaqabeGadaaakeaacqWGybawdaqhaaWcbaGaemyAaKgabaGaemizaqgaaOGaeyipaWJaemiwaG1aa0baaSqaaiabdQgaQbqaaiabdsgaKbaaaaa@35E0@ and as Class *B *otherwise. Advantages of the TSP classifier are the fact that no Parameters need to be estimated (no inner cross-validation is needed), and that the classifier does not suffer from monotonic transformation of the datasets, e.g. data normalization techniques. Formally, *θ*_Ω _and *θ*_Φ _are obsolete, *ϕ *represents the best pair of genes.

### Training and evaluation framework

**Figure 1:**
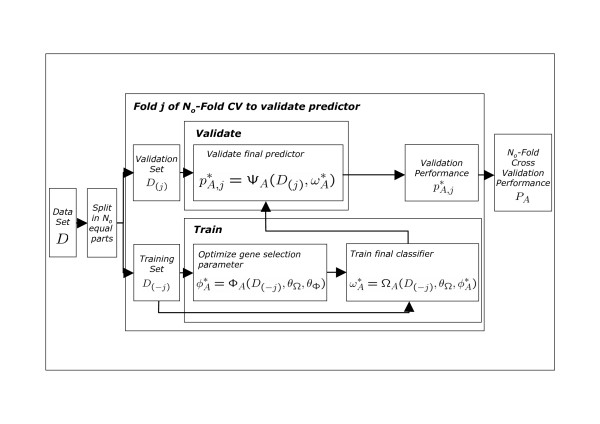
The training-validation protocol employed to evaluate various gene selection and classification approaches in simplified schematic format. The input is a labeled dataset, D, and the Output is an estimate of the validation performance of algorithm *A*, denoted by *P*_*A *_The most important steps in the protocol are the training step (Block labeled 'Train') and the validation step (Block labeled 'Validate'). The training step, in turn, consists of two steps, namely 1) the optimization of the gene selection parameter, *ϕ*, employing a *N*_*i *_– fold cross validation loop and 2) training the final classifier glven the optimal setting of the selection parameter. The validation step estimates the performance of the optimal trained classifier ((ωA*)
 MathType@MTEF@5@5@+=feaafiart1ev1aaatCvAUfKttLearuWrP9MDH5MBPbIqV92AaeXatLxBI9gBaebbnrfifHhDYfgasaacH8akY=wiFfYdH8Gipec8Eeeu0xXdbba9frFj0=OqFfea0dXdd9vqai=hGuQ8kuc9pgc9s8qqaq=dirpe0xb9q8qiLsFr0=vr0=vr0dc8meaabaqaciaacaGaaeqabaqabeGadaaakeaadaqadaqaaGGaciab=L8a3naaDaaaleaacqWGbbqqaeaacqGGQaGkaaaakiaawIcacaGLPaaaaaa@3227@) on the completely independent validation set.

In order to avoid any bias, the selection of the genes and training of the final classifier on the one hand and the evaluation of the classification performance on the other, must be carried out on two independent datasets. To this end, the framework formalized in [29], is adopted here. The framework is graphically depicted in Figure 1. The whole procedure is wrapped in an outer cross-validation loop. (The inner loop will be defined shortly). For *N*_*o*_-fold outer cross validation, the dataset, *D*, is split in *N*_*o *_equally sized and stratified parts. During each of the outer cross validation folds, indexed by *j*, the training set, *D*_(-*j*) _consists of all but the *j*^*th *^part, while the *j*^*th *^part constitutes the validation set, denoted by *D*_(*j*)_- During the training phase, two steps are performed. First, gene selection is performed by optimizing the associated Parameter (Equation 1). This process also employs an *N*_*i*_-fold cross-validation loop (the inner loop) to generate and evaluate gene sets. Each inner fold provides the error curve of the classifier as a function of the number of genes. We compute the average of the curves across the folds. The number of genes that minimizes the average error is considered to define the optimal gene size. Subsequently the classifier is trained on the training set with the optimal parameter setting as input (Equation 3), e.g. the optimal gene size for the glven classifier. The performance of this classifier is only then evaluated on the validation set:

pA,j*=ΨA(D(j),ωA*),     (4)
 MathType@MTEF@5@5@+=feaafiart1ev1aaatCvAUfKttLearuWrP9MDH5MBPbIqV92AaeXatLxBI9gBaebbnrfifHhDYfgasaacH8akY=wiFfYdH8Gipec8Eeeu0xXdbba9frFj0=OqFfea0dXdd9vqai=hGuQ8kuc9pgc9s8qqaq=dirpe0xb9q8qiLsFr0=vr0=vr0dc8meaabaqaciaacaGaaeqabaqabeGadaaakeaacqWGWbaCdaqhaaWcbaGaemyqaeKaeiilaWIaemOAaOgabaGaeiOkaOcaaOGaeyypa0JaeuiQdK1aaSbaaSqaaiabdgeabbqabaGccqGGOaakcqWGebardaWgaaWcbaGaeiikaGIaemOAaOMaeiykaKcabeaakiabcYcaSGGaciab=L8a3naaDaaaleaacqWGbbqqaeaacqGGQaGkaaGccqGGPaqkcqGGSaalcaWLjaGaaCzcamaabmaabaGaeGinaqdacaGLOaGaayzkaaaaaa@45C3@

where pA,j*
 MathType@MTEF@5@5@+=feaafiart1ev1aaatCvAUfKttLearuWrP9MDH5MBPbIqV92AaeXatLxBI9gBaebbnrfifHhDYfgasaacH8akY=wiFfYdH8Gipec8Eeeu0xXdbba9frFj0=OqFfea0dXdd9vqai=hGuQ8kuc9pgc9s8qqaq=dirpe0xb9q8qiLsFr0=vr0=vr0dc8meaabaqaciaacaGaaeqabaqabeGadaaakeaacqWGWbaCdaqhaaWcbaGaemyqaeKaeiilaWIaemOAaOgabaGaeiOkaOcaaaaa@3266@ represents the performance of the optimal classifier on the outer loop validation set of fold *j*, and Ψ_*A*_(.) the function mapping the dataset and classifier to a performance. Averaging the validation performance across the *N*_*o*_folds yields the *N*_*o*_-fold outer cross validation performance of the gene selection technique with the specific user-defined choices. We adopted 10-fold cross-validation for both the inner and outer loops. This choice is suggested by Kohavi [38], and was also applied to gene expression data by Statnikov et al. [7]. The latter obtained similar results using a 10-fold or leave-one-out cross-validation. The former is preferable due to lower computational requirements and lower variance. To estimate the performance of a classification System we use the balanced average classification error which applies a correction for the dass prior probabilities, if these are unbalanced. In this way the results are not dependent on unbalanced classes, and the results on different classifiers can be better compared. The algorithms were implemented in Matlab employing the PRTools [39] and PRExp [40] toolboxes.

### Datasets

In total we employed seven microarray gene expression datasets. Four datasets, *Central Nervous System *(CNS) [41], *Colon *[31], *Leukemia *[8] and *Prostate *[42], were measured on high-density oligonucleotide Affymetrix arrays. Three datasets, *Breast Cancer *[36,43], Diffuse Large B-cell Lymphoma *(DLBCL] *[44] and *Head and Neck Squamous Cell Carcinomas *(*HNSCC*) [45] were hybridized on two-color cDNA platforms. The datasets represent a wide range of cancer types. The tasks are (sub)type prediction (*Colon, Leukemia, DLBCL *and *Prostate*)while for the remaining problems the goal is to predict the future development of the disease: patient survival (*CNS*), probability of future metastasis (*Breast Cancer*)and lymph node metastasis (*HNSCC*).

The *Breast Cancer *dataset consists of 145 lymph node negative breast carcinomas, 99 from patients that did not have a metastasis within five years and 46 from patients that had metastasis within five years. The number of genes is 4919. The *CNS; *dataset is a subset of a larger study. It considers the outcome (survival) after embryonic treatment of the central nervous System. The number of genes is 4458, while the number of samples is 60, divided into 21 patients that survived and 39 that died. The *Colon *dataset is composed of 40 normal healthy samples and 22 tumor samples in a 1908 dimensional feature space. The *DLBCL *dataset is a subset of a larger study which contains measurements of two distinct types of diffuse large B-cell lymphoma. The number of genes is 4026. The total number of samples is 47, 24 belong to the 'germinal center B-like' group while 23 are labeled as 'activated B-like' group. The *Leukemia *dataset contains 72 samples from two types of leukemia where 3571 genes are measured for each sample. The dataset contains 25 samples labeled as acute myeloid (AML) and 47 samples labeled as acute lymphoblastic leukemia (ALL). The *Prostate *cancer dataset is composed of 52 samples from patients with prostate cancer and samples from 50 normal tissue. The number of genes is 5962. For the *HNSCC *dataset, the goal is to predict, based on the gene expression in a primary *HNSCC *tumor, whether a lymph node metastasis will occur. This dataset consists of 66 samples (39 which did metastasize, and 27 that remained disease-free) and the expression of 2340 genes.

The datasets present a variety of the tissue types, technologies and diagnostic tasks. In addition, the panel of sets contains relatively simple, clinically less relevant tasks, such as distinguishing between normal and tumor tissue, as well as more difficult tasks, such as predicting future events based on current samples. We therefore consider the datasets suitable to perform a comparative investigation between univariate and multivariate gene selection techniques.

## Authors' contributions

CL, MJTR and LFAW designed the experiments and analyzed the results; CL carried out the analysis; LJV provided the *Breast Cancer *dataset; all authors participated in the writing of the manuscript.

## Figures and Tables

**Table 1:** The mean and the Standard deviation of the 10-fold cross-validation error (in percentage) for the different approaches and the Affymetrix platform datasets employed in the study.

Method	CNS	Colon	Leukemia	Prostate
Gene selection	mean ± std	mean ± std	mean ± std	mean ± std
U, SNR, NMC	30.4 ± 6.5 *	12.9 ± 4.2 *	4.8 ± 2.7 *	9.7 ± 4.2 *
U, SNR, FLD	42.5 ± 7.3	19.2 ± 5.9	8.0 ± 3.2	10.0 ± 3.0 *
U, t-test, NMC	32.5 ± 4.9 *	12.5 ± 4.2 *	4.8 ± 2.7 *	10.8 ± 3.4
U, t-test, FLD	35.8 ± 6.5 *	11.7 ± 3.5 *	12.0 ± 4.2	8.0 ± 2.5 *

BP greedy, FLD	43.8 ± 6.2	12.9 ± 3.8 *	11.6 ± 3.6	9.8 ± 3.3 *
FS, FLD	47.9 ± 5.1	15.4 ± 4.1	10.2 ± 4.2	14.0 ± 3.4
RFE, FLD	34.2 ± 5.0 *	22.9 ± 4.4	3.5 ± 2.6 *	10.0 ± 2.6 *
RFE, SVM	35.4 ± 5.0 *	22.1 ± 3.5	4.5 ± 2.6 *	8.0 ± 2.9 *
Liknon	32.9 ± 6.1 *	13.3 ± 4.2 *	11.8 ± 4.0	10.8 ± 3.7
TSP	47.0 ± 5.6	5.4 ± 2.9 *	10.6 ± 3.8	7.0 ± 2.6 *

no gene selection	mean ± std	mean ± std	mean ± std	mean ± std
NMC	42.1 ± 5.5	17.9 ± 3.3	3.5 ± 2.6 *	33.7 ± 3.9
FLD	32.9 ± 6.3 *	21.7 ± 3.7	4.5 ± 2.6 *	8.0 ± 2.5 *
SVM	35.4 ± 7.0 *	22.1 ± 3.5	3.5 ± 2.6 *	8.0 ± 2.9 *

**Table 2:** The mean and the Standard deviation of the 10-fold cross-validation error (in percentage) for the different approaches and the cDNA platform datasets employed in the study.

Method	DLBCL	HNSCC	Breast
gene selection	mean ± std	mean ± std	mean ± std
U, SNR, NMC	2.5 ± 2.5 *	21.2 ± 7.1 *	33.0 ± 3.4 *
U, SNR, FLD	15.8 ± 6.4	33.3 ± 6.6	29.9 ± 3.6 *
U, t-test, NMC	2.5 ± 2.5 *	21.2 ± 7.3 *	33.5 ± 3.8 *
U, t-test, FLD	15.8 ± 6.4	36.2 ± 6.2	32.6 ± 3.0 *

BP greedy, FLD	10.0 ± 4.3	36.2 ± 7.0	35.8 ± 2.3
FS, FLD	10.8 ± 3.7	45.4 ± 8.5	35.4 ± 4.2
RFE, FLD	16.7 ± 5.3	35.0 ± 6.3	33.8 ± 3.5
RFE, SVM	15.8 ± 5.2	35.4 ± 7.2	32.6 ± 3.2 *
Liknon	13.3 ± 5.3	37.5 ± 7.4	34.5 ± 5.2
TSP	27.5 ± 2.8	37.6 ± 6.0	49.9 ± 4.6

no gene selection	mean ± std	mean ± std	mean ± std
NMC	6.7 ± 3.5	29.2 ± 7.2	36.7 ± 3.2
FLD	14.2 ± 5.4	32.5 ± 6.6	35.8 ± 4.1
SVM	9.2 ± 3.8	29.6 ± 5.7	34.3 ± 4.2
